# From diagnosis to deadlifts: navigating exercise with type 1 diabetes

**DOI:** 10.1016/j.eclinm.2024.102687

**Published:** 2024-06-20

**Authors:** Alex St. John

**Affiliations:** Institute of Translational Medicine, University of Birmingham, Birmingham, UK

My journey with Type 1 Diabetes (T1D) started in January of 2013. I had just started the second semester of my Human Kinetics degree at the University of Ottawa (in Ottawa, Canada). And while there’s never a great time to be diagnosed with a chronic condition like T1D, it has been one of the true motivators in my academic and athletic careers.

I was in a unique position where I was learning about human performance (in people with and without chronic conditions). And I was able to directly apply that knowledge to my own disease outcomes and athletic performance. My successes (and failures) in translating knowledge from the classroom to real-world situations are the main drivers in my belief that clinician and patient education can truly impact the lives of those living with T1D.

My experiences with clinicians have been both positive and negative. My first endocrinologist in Canada also completed an undergraduate degree in Kinesiology, so they were specifically equipped to handle my questions about exercise and sport as they pertained to T1D. They supported me in my athletic endeavours and understood the complexities of being active with T1D. However, another endocrinologist I saw lacked the knowledge or resourcefulness that I required during my clinical visits when I would discuss sports and exercise. I would leave my appointments disheartened that a clinician would suggest something like bariatric surgery when mentioning my “out-of-control weight gain”, when I was purposefully gaining weight to coincide with gaining strength and improving my athletic performance. When looking back on those negative interactions, I can’t help but feel disappointed at the lack of support for and understanding of my goals by a clinician.

Fuelled by my motivations in all things exercise and sport, I wanted to learn more. The logical next step was more education. My post-graduate degree was in Diabetes Medicine at the University of Dundee, where I sought to determine barriers and facilitators of regular physical activity in those with T1D. With regular exercise or sport being such a focal point of my disease outcomes, I wanted to find out why people with T1D aren’t as active as people without.^1^, ^2^, ^3^

Some of the main barriers to exercise include fear of hypoglycaemia, loss of control over T1D, low fitness level, overall lack of knowledge related to exercise and T1D, and lack of social support.^4^^,^^5^ What I often hear from people with T1D is that they don’t feel as though they could add exercise to their lives because so much of the condition is trial-and-error already. Even without T1D, exercising can present as such a great challenge, never mind the continuous thought of a looming “hypo” on the mind of someone with T1D. Worrying about adequate carb intake before, during, and after exercise; insulin dosages or pharmacokinetics; and the duration, intensity, and timing of exercise, can all be too much! But it doesn’t have to be.

I believe that many of the barriers to exercise or sport in T1D can be attributed to a knowledge gap in those with T1D. From conversations with other people with T1D over the years, I’ve found that many of them don’t know where to find resources on exercise, or what questions to ask their clinicians. Their clinicians may not know the answers to a very specific issue a person with T1D is experiencing related to a specific physical activity they’ve just begun. Without adequate knowledge of how to exercise with T1D, it will be difficult to feel confident to exercise. Those with T1D should be able to find the information they need to exercise safely, and a large part of that challenge can be met with online resources like peer support groups or science communication on social media.

There are so many tremendous research efforts around the globe working on projects like refining exercise guidelines, further elucidating the body’s responses to different modalities of exercise, or using different types of technology to make exercising with T1D that much easier. But the average person with T1D may not know how to access these journal articles, or how to decipher the technical jargon that’s often used to explain the results of any particular study. Knowledge translation, particularly done by science communicators, is one of the crucial pathways for those with T1D to actually use clinically relevant research findings in their athletic endeavours. Discussions involving exercise or sport and those with T1D in online and clinical settings need to be based on a strong foundation of evidence; summarising clinically relevant and statistically significant findings, and improving general access to those findings are great steps towards that reality.

## Contributors

ASJ is the sole contributor to the writing of this Commentary.

## Declaration of interests

ASJ previously held an independent contractor position with The Diabetes Link as a Content Creator; received travel, accommodation, and conference registration fees for Diabetes UK Professional Conference 2024 from #dedoc°; and received travel, accommodation, and conference registration for ADCES Conference 2023 from The Diabetes Link.
